# Bacterial membrane vesicles of *Pseudomonas aeruginosa* activate adenosine monophosphate-activated protein kinase signaling through inhibition of mitochondrial complex III

**DOI:** 10.1093/pnasnexus/pgaf248

**Published:** 2025-08-19

**Authors:** Julia Müller, Marcel Kretschmer, Elise Opitsch, Svea Holland, José Manuel Borrero-de Acuña, Dieter Jahn, Meina Neumann-Schaal, Andre Wegner

**Affiliations:** Braunschweig Integrated Centre of Systems Biology (BRICS), Technische Universität Braunschweig, Rebenring 56, 38106 Braunschweig, Germany; Department of Bioinformatics and Biochemistry, Technische Universität Braunschweig, Rebenring 56, 38106 Braunschweig, Germany; Braunschweig Integrated Centre of Systems Biology (BRICS), Technische Universität Braunschweig, Rebenring 56, 38106 Braunschweig, Germany; Department of Bioinformatics and Biochemistry, Technische Universität Braunschweig, Rebenring 56, 38106 Braunschweig, Germany; Braunschweig Integrated Centre of Systems Biology (BRICS), Technische Universität Braunschweig, Rebenring 56, 38106 Braunschweig, Germany; Department of Bioinformatics and Biochemistry, Technische Universität Braunschweig, Rebenring 56, 38106 Braunschweig, Germany; Braunschweig Integrated Centre of Systems Biology (BRICS), Technische Universität Braunschweig, Rebenring 56, 38106 Braunschweig, Germany; Leibniz Institute DSMZ, German Collection of Microorganisms and Cell Cultures GmbH, Inhoffenstraße 7B, 38124 Braunschweig, Germany; Departamento de Microbiología, Facultad de Biología, Universidad de Sevilla, Av. de la Reina Mercedes 6, 41012 Sevilla, Spain; Braunschweig Integrated Centre of Systems Biology (BRICS), Technische Universität Braunschweig, Rebenring 56, 38106 Braunschweig, Germany; Institute for Microbiology, Technische Universität Braunschweig, Rebenring 56, 38106 Braunschweig, Germany; Braunschweig Integrated Centre of Systems Biology (BRICS), Technische Universität Braunschweig, Rebenring 56, 38106 Braunschweig, Germany; Leibniz Institute DSMZ, German Collection of Microorganisms and Cell Cultures GmbH, Inhoffenstraße 7B, 38124 Braunschweig, Germany; Braunschweig Integrated Centre of Systems Biology (BRICS), Technische Universität Braunschweig, Rebenring 56, 38106 Braunschweig, Germany; Department of Bioinformatics and Biochemistry, Technische Universität Braunschweig, Rebenring 56, 38106 Braunschweig, Germany

**Keywords:** bacterial membrane vesicles (BMVs), *Pseudomonas aeruginosa*, metabolism, electron transport chain, AMPK

## Abstract

Bacterial membrane vesicles (BMVs) are secreted by many pathogenic bacteria and known to stimulate various host responses upon infection, thereby contributing to the pathogenicity of bacterial pathogens like *Pseudomonas aeruginosa*. While the effects of BMVs on host immune responses are well studied, little is known about their impact on cell metabolism and mitochondrial respiration. Here, we show that *P. aeruginosa* BMVs (i) reprogram cell metabolism of human lung cells, (ii) negatively affect mitochondrial respiration by (iii) specifically inhibiting complex III of the electron transport chain, leading to (iv) the activation of adenosine monophosphate-activated protein kinase (AMPK) signaling, which in turn results in (v) AMPK-dependent inhibition of global protein synthesis.

Significance Statement The study reveals that *Pseudomonas aeruginosa* bacterial membrane vesicles (BMVs) significantly disrupt human lung cell metabolism and mitochondrial function. Specifically, BMVs inhibit complex III of the electron transport chain, leading to impaired mitochondrial respiration. This disruption activates adenosine monophosphate-activated protein kinase (AMPK) signaling, which subsequently results in an AMPK-dependent inhibition of global protein synthesis. These findings elucidate a previously uncharacterized mechanism by which BMVs contribute to bacterial pathogenicity.

## Introduction

The opportunistic pathogen *Pseudomonas aeruginosa* causes milder local to severe systemic infections. These infections by often multiantibiotic resistant bacteria are particularly prevalent in immunocompromised patients, emphasizing its importance as a significant health concern in global health care (1, 2). During infection, *P. aeruginosa* secretes bacterial membrane vesicles (BMVs) containing metabolites, nucleic acids, and proteins, including virulence factors, which are delivered to the host cell during infection and play a pivotal role in its pathogenicity (3–6). BMVs are well known for triggering host immune responses, capable of inducing the release of proinflammatory cytokines like interleukin-8 (7–9). Notably, BMVs can also activate the adenosine monophosphate-activated protein kinase (AMPK) within the host cell, leading to autophagy induction (10). However, how the activation of AMPK in response to BMVs is regulated remains unclear.

Given that AMPK serves as a metabolic sensor, activated by mitochondrial dysfunction (11, 12), and considering evidence of BMVs inhibiting mitochondrial activity in macrophages (13), it is conceivable that mitochondrial dysfunction might be the primary event facilitating BMV-induced AMPK activation. Some bacterial pathogens are known to affect mitochondrial function by specifically inhibiting protein complexes of the electron transport chain (ETC), which is essential for oxidative phosphorylation and ATP generation (14). For *P. aeruginosa*, extracellular secreted factors such as 1-hydroxyphenazine, pyocyanin, and exotoxin A have been described as possible inhibitors of the ETC, leading to reduced mitochondrial respiration (15–18). However, the functional basis of mitochondrial dysfunction caused by BMVs is still unknown.

Here, we show that treatment of human lung cells with BMVs isolated from the pathogenic *P. aeruginosa* strain PA14 leads to metabolic reprogramming and impaired mitochondrial respiration by specifically inhibiting complex III of the ETC, resulting in mitochondrial dysfunction. Moreover, this event activates AMPK signaling, leading to AMPK-dependent inhibition of global protein synthesis in the host cell.

## Materials and methods

### Bacterial strains and isolation of BMVs

The bacterial strains *P. aeruginosa* PA14 (19) and *Pseudomonas putida* KT2440 (20) were used in this study. Both strains were cultivated in lysogeny broth (LB) (Roth, X968.2) in flasks with baffles. BMVs were isolated as described before (21). Briefly, bacterial cultures were inoculated with an optical density measured at a wavelength of 600 nm (OD_600_) of 0.05 and grown at 37 °C (*P. aeruginosa*) or 30 °C (*P. putida*) at 160 rpm until they reached the early-stationary phase of growth. BMVs were isolated from culture media after removing bacterial cells by centrifugation at 4 °C and 8,000*×g* for 30 min and filtration through 0.22-µM PES filters (Corning, 431097). Supernatants were concentrated by ultrafiltration using Vivaspin 20 PES ultrafiltration units with a molecular weight cutoff of 100 kDa (Sartorius, VS2041). BMVs were isolated by ultracentrifugation at 4 °C and 150,000*×g* for 2 h. Isolated vesicles were resuspended in 1 × PBS (Gibco, 18912-014) and sterile-filtered. BMVs were quantified by using the membrane lipid dye FM4-64 (Invitrogen, T13320) to calculate the vesicle load per µL (VL/µL) as described before (21). Vesicle samples were stored at 4 °C.

### Cell culture

The cell lines A549 (DSMZ, ACC 107), HCC44 (DSMZ, ACC 534), and human bronchial epithelial cells (HBEpC, PromoCell, C-12640) were used in this study. A549 cells were cultivated in Dulbecco's modified Eagle medium (DMEM) (Gibco, 11965-092) containing 25 mM glucose and 4 mM glutamine. HCC44 cells were cultivated in RPMI 1640 medium (Gibco, 21875-034) containing 11 mM glucose and 2 mM glutamine. Both growth media were supplemented with 10% FBS (Bio&SELL, FBS.SAM.0500). HBEpC cells were cultivated in Airway Epithelial Cell Growth Medium supplemented with Growth Medium SupplementMix (PromoCell, C-21060). All cell lines were incubated in a humidified atmosphere with 5% CO_2_ at 37 °C. Cell detachment was performed with 0.05% trypsin–EDTA (Gibco, 25300-054) for A549 and HCC44 cells or accutase solution (Sigma-Aldrich, A6964) for HBEpC cells.

### Analysis of cell growth and viability

To analyze cell growth of the proliferating cell lines A549 and HCC44, cell confluence was measured in 6-well plates (Greiner Bio-One, 657160) at 37 °C with a microplate reader (Tecan Spark). For the analysis of cell viability of nonproliferating HBEpC cells, the PrestoBlue cell viability reagent (Invitrogen, A13261) was used according to the user manual. Fluorescence signals were measured by using an excitation wavelength of 560 nm and an emission wavelength of 590 nm.

### Stable isotope labeling

For stable isotope labeling, cells were seeded in 6-well plates and incubated at 37 °C and 5% CO_2_ overnight. The next day, cells were washed with 1 × PBS and treated as indicated in the figure legends in the following labeling media: A549 cells were cultivated in DMEM (Gibco, A14430-01) supplemented with 25 mM unlabeled glucose or [U-^13^C_6_]-glucose and 4 mM [U-^13^C_5_]-glutamine or unlabeled glutamine and 10% dialyzed FBS (dFBS). HCC44 cells were cultivated in SILAC RPMI 1640 medium (Gibco, A24942-01) supplemented with 11.1 mM unlabeled glucose or [U-^13^C_6_]-glucose, 2.05 mM [U-^13^C_5_]-glutamine or unlabeled glutamine, 0.22 mM lysine, 1.15 mM arginine, and 10% dFBS. HBEpC cells were cultivated in DMEM (Gibco, A14430-01) supplemented with 25 mM unlabeled glucose or [U-^13^C_6_]-glucose, 4 mM [U-^13^C_5_]-glutamine or unlabeled glutamine, and 2% growth medium SupplementMix (PromoCell, C-39165).

### Metabolite extraction and gas chromatography coupled with mass spectrometry analysis

For gas chromatography coupled with mass spectrometry (GC/MS) analysis of cellular metabolites, cells were seeded in 6-well plates and incubated at 37 °C and 5% CO_2_ overnight. The next day, cells were washed with 1 × PBS and treated as indicated in the figure legends. Treated cells were extracted as described by Sapcariu et al. (22). The upper polar phase was transferred in GC/MS vials and dried in a speedvac at 4 °C overnight. Dried samples were derivatized with 15 µL of 2% (w/v) methoxyamine hydrochloride solved in pyridine by shaking at 40 °C for 90 min and additional 15 µL of *N*-methyl-*N*-(trimethylsilyl)trifluoroacetamide (MSTFA) by shaking at 40 °C for 30 min or *N*-*tert*-butyldimethylsilyl-*N*-methyltrifluoroacetamide (MTBSTFA) by shaking at 55 °C for 60 min.

The derivatized samples (1 µL) were injected into a SSL injector in splitless mode and heated up to 270 °C. GC/MS measurements were taken with an Agilent Technologies 7890B GC system including a 30-m Phenomenex ZB-35 and a 5-m Duraguard capillary column, connected to an Agilent Technologies 5977B MSD, under electron ionization at 70 eV. The MS source temperature was held at 230 °C and the quadrupole temperature at 150 °C. Helium was used as a carrier gas with a flow rate of 1 mL/min. The temperature profile of the GC oven depended on the used measuring method. For the measurement of MTBSTFA-derivatized samples, the GC oven temperature was held at 100 °C for 2 min, then increased up to 300 °C at 10 °C/min, and held at 300 °C for 4 min. For the measurement of MSTFA-derivatized samples, the GC oven temperature was held at 80 °C for 6 min, then increased up to 300 °C at 6 °C/min, held at 300 °C for 10 min, raised to 325 °C at 10 °C/min, and held at 325 °C for 4 min. The total abundances of metabolites and distributions of mass isotopomers were calculated by the integration of mass fragments and corrected for natural isotope abundances by using the software MetaboliteDetector as previously described (23).

### Quantitative PCR analysis

RNA was isolated from the interphase of extracted cells as described before (22) by using the NucleoSpin RNA kit (MACHEREY-NAGEL, 740955.50). Isolated RNA was converted to cDNA by using the High-Capacity cDNA Reverse Transcription Kit (Applied Biosystems, 4368813). RNA and cDNA concentrations were measured with a microplate reader (Tecan Spark). For qPCR analysis, TaqMan gene expression assays (Applied Biosystems) for the housekeeping gene 18S (Hs99999901 s1) and target genes HMGCR (Hs00168352 m1) and SREBF2 (Hs01081784 m1) were used, together with the iTaq Universal Probes Supermix (Bio-Rad, 1725132) and the QuantStudio 5 Real-Time PCR system (Applied Biosystems). Data were analyzed with the QuantStudio design and analysis software.

### Protein extraction and immunoblotting

For protein analysis, cells were plated in 10-cm (A549 and HCC44 cells) or 6-cm (HBEpC cells) cell culture dishes (Greiner Bio-One) in growth medium and incubated at 37 °C and 5% CO_2_ overnight. The next day, cells were treated as indicated in the figure legends. After treatment, cells were lysed with the M-PER extraction reagent (Thermo Scientific, 78501) containing 1 × Halt protease and phosphatase inhibitors (Thermo Scientific, 78441), mixed at 4 °C and 1,400 rpm for 10 min, and centrifuged at 4 °C and 14,000*×g* for 10 min. Supernatants containing protein were stored at −20 °C. Protein quantification was performed by using the Pierce BCA protein assay kit (Thermo Scientific, 23227) according to the user manual. Samples were loaded with a 5 × Laemmli buffer, incubated at 95 °C for 5 min, and centrifuged at 16,000*×g* for 1 min. Twenty-five micrograms of total protein were separated on 4–20% precast SDS–PAGE gels (Bio-Rad). Depending on the protein size, a prestained protein marker covering the range of 10–180 kDa (Thermo Scientific, 26616) or 43–315 kDa (Cell Signaling Technology, 12949) was used.

Proteins were transferred onto a 0.45-µm PVDF membrane (Carl Roth, T830.1) by using the Trans-Blot SD Semi-Dry Transfer Cell (Bio-Rad). The membrane was blocked with 5% BSA (w/v) (Biomol, 01400.100) in Tris-buffered saline with 0.1% Tween 20 (TBS-T) for 1 h. Primary antibodies diluted in 1 × TBS-T with 5% BSA (Table S1) were added to the membranes and incubated at room temperature for 1 h. Membranes were then incubated with secondary antibodies diluted in 1 × TBS-T with 5% BSA (Table S2) at room temperature for 1 h. For signal detection, the Immobilon Classico Western HRP substrate (Millipore, WBLUC0500) was used and imaged with a Bio-Rad ChemiDoc imaging system. Band intensities were analyzed by using the ImageJ 1.53k software.

### Respirometry

Cell respiration was measured by using an Agilent Seahorse XFe96 Analyzer together with Seahorse XFe96 Extracellular Flux Assay Kits. Cells were seeded in Seahorse XF96 V3 PS Cell Culture Microplates (Agilent, 101085-004) and cultured in growth medium at 37 °C and 5% CO_2_ overnight. At the same time, the sensor cartridge (Agilent, 103792-100) was incubated with sterile water together with the Seahorse XF Calibrant Solution (Agilent, 100840-000) at 37 °C. The next day, the medium was replaced with Seahorse XF DMEM medium, pH 7.4 (Agilent, 103575-100), supplemented with 10 mM glucose, 2 mM glutamine, and 10% dFBs and incubated at 37 °C for 60 min. For sensor cartridge calibration, the water was replaced with Seahorse XF Calibrant Solution and incubated at 37 °C for 45 min. To assess mitochondrial function, the oxygen consumption rate (OCR) (pmol O_2_/min) was measured and normalized to basal respiration. Mitochondrial respiratory chain deficiencies were analyzed based on the study of Jaber et al. (24).

## Results and discussion

### *P. aeruginosa* BMVs inhibit proliferation and reduce viability of human lung cells

**Fig. 1.**
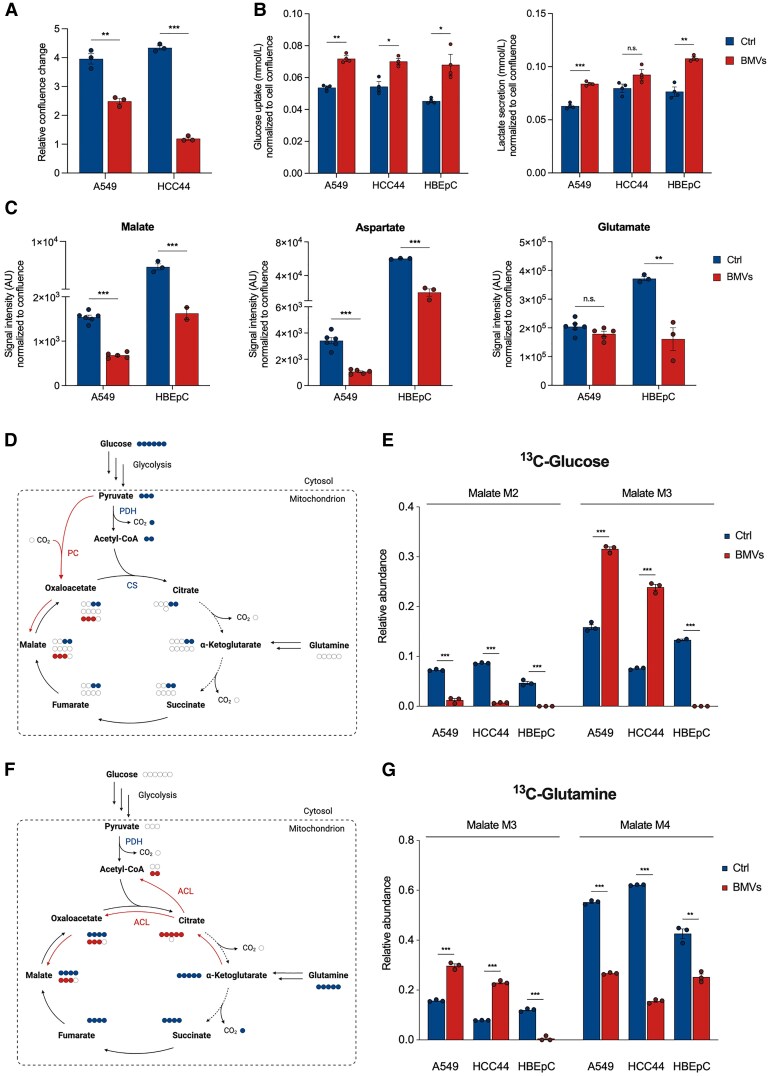
Metabolic reprogramming in host cells after PA14 BMV treatment. A) Confluence of A549 and HCC44 cells after treatment with 25 VL/mL BMVs for 72 h. Data were obtained from three replicates. B) Glucose uptake and lactate secretion of A549, HCC44, and HBEpC cells after treatment with 25 VL/mL BMVs for 24 h. Data were obtained from three replicates. C) Signal intensities (AU) of malate, aspartate, and glutamate in A549 and HBEpC cells after vesicle treatment (25 VL/mL for 24 h). Data were obtained from 5 to 6 (A549) or 2 to 3 (HBEpC) replicates and normalized to cell confluence. D) Schematic overview of the incorporation of a [U-^13^C_6_]-glucose tracer into metabolites of the TCA cycle. Created with BioRender.com. E) Malate MIDs after [U-^13^C_6_]-glucose labeling of BMV-treated cells. A549 and HCC44 cells were treated for 24 h and HBEpC cells for 6 h with 25 VL/mL. Data were obtained from two or three replicates. F) Schematic overview of the incorporation of a [U-^13^C_5_]-glutamine tracer into metabolites of the TCA cycle. Created with BioRender.com. G) Malate MIDs after [U-^13^C_5_]-glutamine labeling of BMV-treated cells. A549 and HCC44 cells were treated for 24 h and HBEpC cells for 6 h with 25 VL/mL. Data were obtained from three replicates. All bar plots in this figure are depicted as mean ± SEM. For the comparison between control (Ctrl) and BMV-treated cells for each cell line, statistical significance was analyzed using unpaired t test (n.s. = not significant, *P < 0.05, **P < 0.01, ***P < 0.001).

BMVs from several bacterial species are reported to inhibit the proliferation of different host cell types (21, 25–27). Thus, we aimed to explore the impact of *P. aeruginosa* BMVs on the proliferation of human lung cells in initial experiments. To this end, we treated A549 and HCC44 lung cancer cells with BMVs isolated from the pathogenic *P. aeruginosa* strain PA14 and monitored their cell confluence over 72 h. Moreover, we analyzed the effects of PA14 BMVs on the viability of primary bronchial epithelial cells (HBEpC) by fluorescently labeling living cells, as these cells are nonproliferating. For all cell lines, we observed a decrease in cell confluence (Fig. 1A) or viability (Fig. S1A) after vesicle treatment, highlighting the pathogenic potential of PA14 BMVs. In contrast, treatment with BMVs isolated from the nonpathogenic strain *P. putida* KT2440 resulted in a significantly weaker effect (Fig. S1A and B). This suggests that the specific cargo of the pathogenic PA14 BMVs was responsible for the host reaction, rather than BMVs in general. To additionally exclude that the effects on cell confluence are not mediated by potential contaminants from vesicle isolation or components of the media and solutions used, we treated A549 cells with a LB medium concentrate prepared under identical conditions as BMVs. Neither the LB concentrate nor *P. putida* KT2440 BMVs significantly affected A549 cell confluence compared with PBS-treated cells, suggesting that the specific cargo of the pathogenic PA14 BMVs was responsible for the host reaction (Fig. S1C). However, the precise molecular mechanism of the observed antiproliferative effect remains unknown.

### *P. aeruginosa* BMVs induce rapid metabolic reprogramming by modulating TCA cycle activity

BMVs have been recognized for modulating the host immune response during *P. aeruginosa* infection (7, 8). However, the underlying molecular dynamics remain elusive. Given the close link between cellular functions and cell metabolism, investigating the metabolic responses of the host cell induced by PA14 BMVs would be revealing for the understanding of the host–pathogen interaction of *P. aeruginosa*. Recently, we developed a method for the isolation and quantification of BMVs in order to analyze vesicle-induced metabolic changes in mammalian cell cultures (21). To ascertain the potential metabolic effects induced by BMVs, we initially examined changes in glucose uptake and lactate secretion of vesicle-treated lung cancer cells as well as primary bronchial epithelial cells. We observed an increase in glucose uptake and lactate secretion for all tested cell lines (Fig. 1B), indicating increased glycolytic activity. This phenomenon is known for various bacterial and viral infections, especially in immune cells, presumably to provide biosynthetic intermediates for the synthesis of nucleotides, amino acids, and lipids to support host cell proliferation (28–30).

To further investigate BMV-induced metabolic shifts in host cells, we employed GC/MS to analyze BMV-treated A549, HCC44, and HBEpC cells. Our results indicate broad metabolic reprogramming of both lung cancer and primary lung cells. Specifically, we observed decreased levels of TCA cycle-associated metabolites such as malate, aspartate, and glutamate in cells treated with PA14 BMVs (Fig. 1C). Analogous to cell growth, these effects were reduced when cells were treated with BMVs of the nonpathogenic *P. putida* KT2440 strain (Fig. S1D and E). Conversely, the levels of most amino acids were increased in A549 cells after BMV treatment, presumably due to autophagy induction, as previously reported (21).

To understand the metabolic pathways contributing to these observations, we used stable isotope-assisted metabolomics. By feeding cells with [U-^13^C_6_]-glucose and [U-^13^C_5_]-glutamine during vesicle exposure, we noted considerable shifts in the mass isotopomer distribution (MID) of TCA cycle-associated metabolites, exemplified by malate. As glucose can enter the TCA cycle via acetyl-CoA, the use of the [U-^13^C_6_]-glucose tracer results in the formation of M2 citrate (Fig. 1D) and, for the oxidative TCA cycle flux, in M2 malate. Interestingly, the formation of M2 malate was decreased in all tested cell lines after vesicle treatment (Fig. 1E). For A549 and HCC44 cells, we also observed an increased formation of M3 malate from [U-^13^C_6_]-glucose, involving the conversion of fully labeled pyruvate to oxaloacetate by carboxylation via pyruvate carboxylase (PC). In this step, free unlabeled CO_2_ gets incorporated into oxaloacetate resulting in M3 malate (Fig. 1D). The increased formation of M3 malate in A549 and HCC44 cells after BMV treatment (Fig. 1E) suggests a shift towards increased PC activity, presumably to compensate for the reduced oxidative TCA cycle flux. However, we did not observe this effect in HBEpC cells, indicating an inactive PC and no possibility to evade the affected flux.

Using [U-^13^C_5_]-glutamine as a substrate, active oxidative TCA cycle flux results in M4 malate (Fig. 1F). After BMV treatment, we observed decreased formation of M4 malate from [U-^13^C_5_]-glutamine for all tested cell lines (Fig. 1G), confirming the ^13^C-glucose results. Moreover, the formation of M3 malate increased in A549 and HCC44 cells (Fig. 1G), explained by a shift to the reductive TCA cycle flux (31), resulting in M5 citrate that can be converted to M2 acetyl-CoA and M3 oxaloacetate via ATP-citrate lyase (ACL, Fig. 1F). Similar to the glucose labeling results, we did not observe the increased formation of M3 malate in HBEpC cells (Fig. 1G). Deregulation of enzymes such as PC and ACL, associated with the reductive TCA cycle flux, is known for various pathological conditions like cancer, but also in infection (32, 33). Our results suggest that the shift towards a higher activity of these enzymes after BMV treatment is specific for lung cancer cell metabolism and does not occur in primary lung cells. To investigate whether these effects are caused by specific virulence agents of PA14 BMVs or vesicles in general, we analyzed potential effects of BMVs isolated from the nonvirulent *P. putida* KT2440 strain. We observed much smaller effects when cells were treated with these vesicles, which further suggests that the effects are due to specific factors contained in BMVs isolated from the pathogenic *P. aeruginosa* PA14 strain (Fig. S1F and G).

Given that *P. aeruginosa* BMVs contain lipopolysaccharide (LPS) on their surface, which is known to induce a potent immune response in the host (8), we analyzed whether LPS mediates similar effects on cell metabolism in A549 cells.

To that end, we performed [U-^13^C_6_]-glucose and [U-^13^C_5_]-glutamine labeling during treatment with 1 µg/mL LPS. We observed no metabolic changes, shown by the similar malate MIDs after LPS treatment compared with the control cells (Fig. S2), excluding LPS as the responsible factor of BMV-mediated metabolic reprogramming.

To analyze how fast the observed metabolic changes appear after BMV treatment, we analyzed the effects after different treatment times (15, 60, and 240 min). We observed rapid metabolic reprogramming following BMV treatment, with decreased M2 and increased M3 malate already observable after 15 min in A549 cells (Fig. S3A). Moreover, the cellular malate levels were reduced by 20% at this time (Fig. S3B). Our results indicate a rapid PA14 vesicle-driven impact on cell metabolism, evidenced by the decreased oxidative TCA cycle flux following BMV treatment. The swift onset of this effect suggests that the observed metabolic changes are not due to prior cellular alterations, such as changes at gene expression level. Instead, this indicates the presence of a vesicle-associated factor that triggers an immediate effect on cellular respiration.

### *P. aeruginosa* BMVs impair mitochondrial respiration of human lung cells by inhibiting ETC complex III

**Fig. 2.**
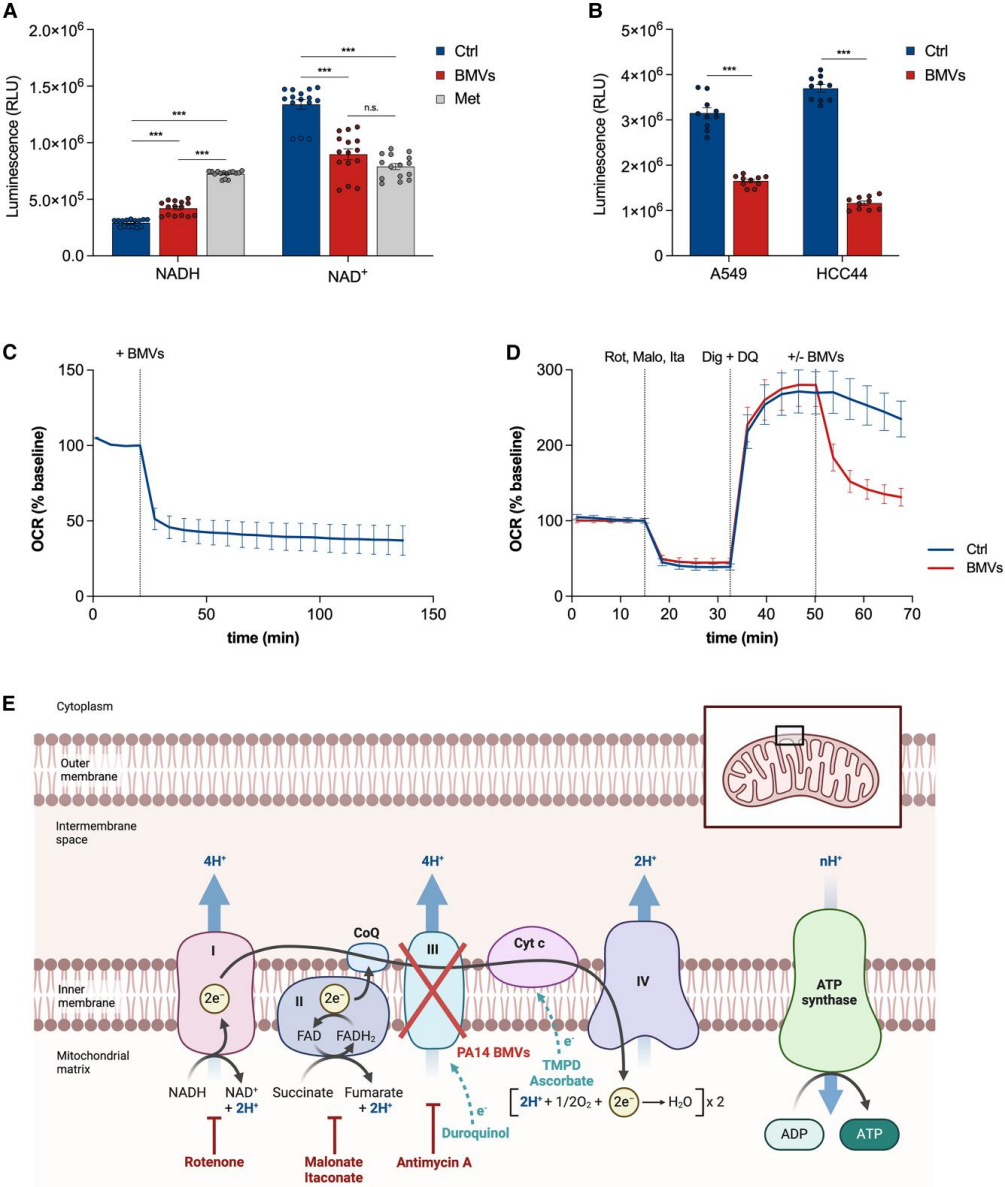
PA14 BMVs induce mitochondrial dysfunction in host cells. A) NADH and NAD^+^ levels of A549 cells after treatment with 25 VL/mL BMVs and 1 mM metformin (Met) for 24 h measured as luminescence intensities (RLU). Data were obtained from 15 replicates. B) ATP levels of A549 and HCC44 cells after BMV treatment (25 VL/mL for 24 h) measured as luminescence intensities (RLU). Data were obtained from 10 replicates. C) OCR of A549 cells after BMV treatment. 25 VL/mL BMVs were added at the depicted time point. Data were obtained from 15 replicates. D) Respirometry test for complex III activity of the ETC of A459 cells after BMV treatment (25 VL/mL). Isolation of complex III was performed by the addition of 2 µM rotenone (Rot), 40 µM malonate (Malo), 2 mM itaconate (Ita), 25 µg/mL digitonin (Dig), and 1 mM duroquinol (DQ) at the depicted time points. Data were obtained from 30 replicates. All OCR values in this figure were normalized to the baseline and depicted as mean ± SEM. E) Location of electron transport deficiencies by functionally isolating cytochrome *c* and the complexes of the ETC to test their activity after vesicle treatment. Rotenone, malonate, and itaconate or AA are added to inhibit complex I, II, or III, which interrupts the electron transfer to the following complexes. The electron transport can be restored by the addition of the electron donor duroquinol for complex III or TMPD and its reducing agent ascorbate for cytochrome *c*. PA14 BMVs are able to specifically inhibit complex III of the ETC. All bar plots in this figure are depicted as mean ± SEM. Statistical significance was analyzed using unpaired one-way ANOVA followed by Tukey's multiple comparison test (A) or using unpaired t test (B) (n.s. = not significant, ***P < 0.001).

The aforementioned metabolic changes indicate a reduced oxidative TCA cycle flux after BMV treatment, resulting in decreased generation of NADH and FADH_2_, which are essential for mitochondrial respiration and ATP production. Deo et al. (13) have previously demonstrated the inhibitory effects of *P. aeruginosa* BMVs on mitochondrial activity in macrophages, leading to mitochondrial apoptosis and inflammation. Consistent with these results, we observed a significantly higher NADH/NAD^+^ ratio and decreased ATP levels in A549 and HCC44 cells after BMV treatment (Fig. 2A and B). To determine whether *P. aeruginosa* BMVs alter mitochondrial respiration in human lung cells, we analyzed changes of cellular respiration by measuring the OCR in BMV-infected A549 cells. We observed a decreased OCR immediately after vesicle treatment (Fig. 2C), indicating a rapid effect on mitochondrial respiration (*<*7 min), which might explain the swift metabolic adaptation discussed above.

Mitochondrial respiration relies on oxidative phosphorylation, which requires the ETC located in the inner mitochondrial membrane and composed of complexes I, II, III, and IV, as well as proteins like cytochrome *c*. It is well known that some bacterial pathogens are able to inhibit complexes of the ETC (14). For *P. aeruginosa*, the extracellular secreted factors exotoxin A, 1-hydroxyphenazine, and pyocyanin have been reported to affect mitochondrial respiration, presumably by disrupting the ETC (15–18). To determine whether *P. aeruginosa* BMVs specifically impair one of the ETC complexes, leading to the observed respiratory deficit, we followed the protocol by Jaber et al. (24). This protocol involves a stepwise series of experiments to map the location of electron transport deficiencies, beginning with cytochrome *c*. If BMVs cause a cytochrome *c* deficit, then adding exogenous cytochrome *c* should rescue respiration. Because cytochrome *c* cannot cross the plasma membrane, we selectively permeabilized the plasma membrane using digitonin. However, we did not observe a rescue effect in the OCR of BMV-treated A549 cells supplemented with cytochrome *c* (Fig. S4A), suggesting that a deficit in another component of the ETC is limiting respiration.

After excluding cytochrome *c* deficiency, we functionally isolated the individual complexes of the ETC by selectively limiting electron entry to each complex and determining whether the deficiency can still be observed. Since complexes I to IV function in series, a dysfunctional complex would affect subsequent complexes. For this reason, we started with complex IV.

To functionally isolate complex IV, we inhibited complex III with antimycin A (AA) (Fig. 2E), which interrupts electron transfer to complex IV, leading to a decreased OCR (Fig. S4B). We then added *N*,*N*,*N*′,*N*′-tetramethyl-*p*-phenylenediamine (TMPD) and its reducing agent ascorbate to restore electron transfer to complex IV, bypassing complex III (Fig. 2E). We observed that complex IV activity was not affected by BMVs (Fig. S4B), leaving complexes I to III as potential targets.

Next, we analyzed complex III activity by inhibiting complex I with rotenone and complex II with malonate and itaconate (Fig. 2E) (34, 35). To ensure electron transfer to complex III, we added the electron donor duroquinol, which recovers respiration by feeding electrons into complex III (Fig. 2E). Interestingly, we observed impaired complex III activity immediately after vesicle addition, indicating specific inhibition of this complex by PA14 BMVs (Fig. 2D). Moreover, we observed impairment of complexes I and II, presumably as a consequence of complex III inhibition (Fig. S4C and D).

Overall, these findings suggest that PA14 BMVs specifically target complex III of the ETC, leading to a cascade of inhibition that affects complexes I and II, thereby impairing overall mitochondrial respiration.

### *P. aeruginosa* BMVs suppress cholesterol biosynthesis and global protein synthesis while inducing inflammation

**Fig. 3.**
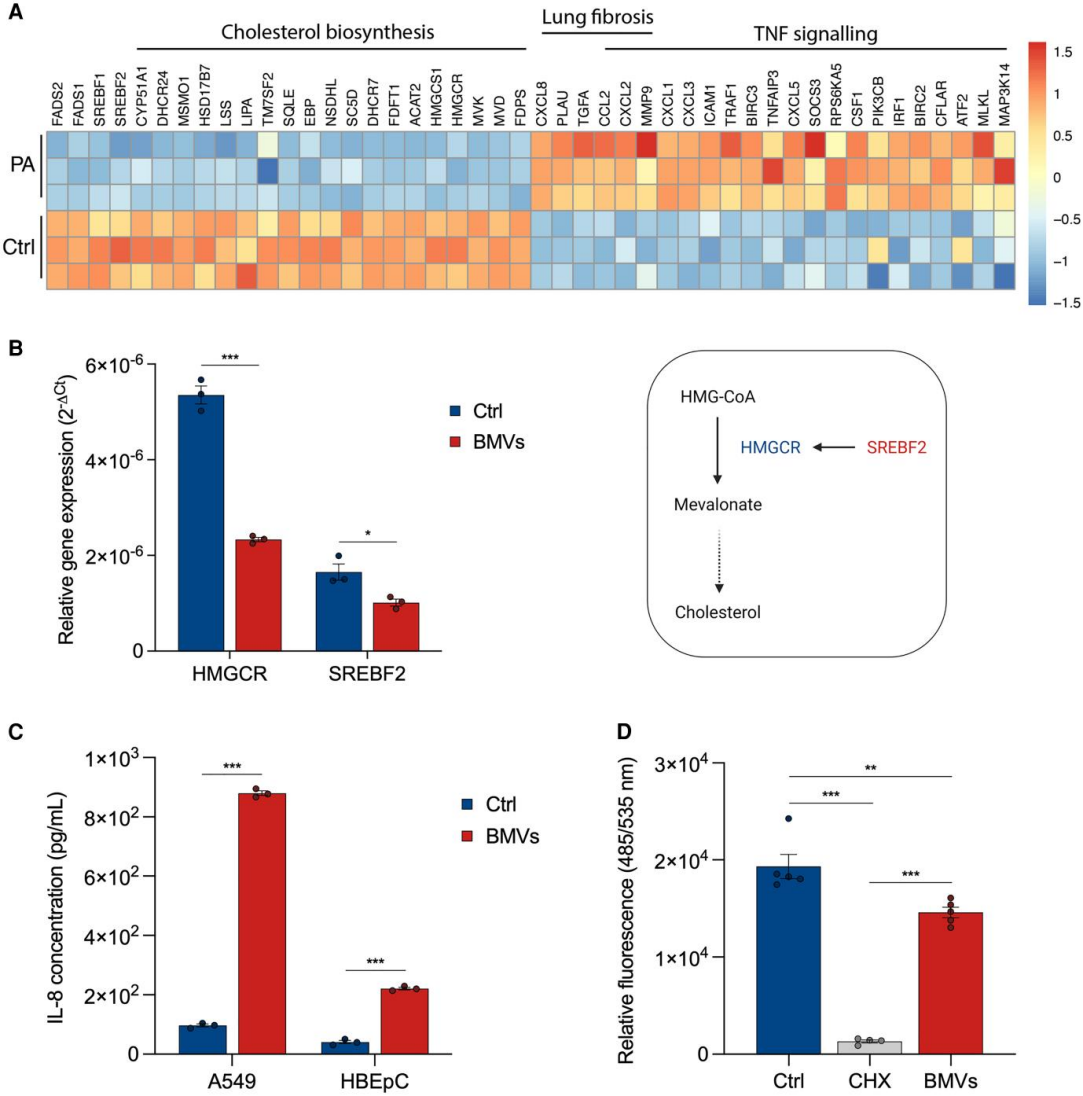
PA14 BMVs affect transcription and translation of the host cell. A) RNA-sequencing analysis of A549 cells treated with PA14 BMVs (PA) for 24 h. B) Relative gene expression of HMGCR and SREBF2 in A549 cells after treatment with 25 VL/mL BMVs for 24 h. Data were obtained from three replicates. C) IL-8 secretion of A549 and HBEpC cells after BMV treatment (25 VL/mL for 24 h). Data were obtained from three replicates. D) Global protein synthesis of A549 cells after treatment with 50 µg/mL CHX and 25 VL/mL BMVs for 30 min (+30 min pretreatment). Data were obtained from four or five replicates. All bar plots in this figure are depicted as mean ± SEM. Statistical significance was analyzed using unpaired t test (B, C) or using unpaired one-way ANOVA followed by Tukey's multiple comparison test (D) (*P < 0.05, **P < 0.01, ***P < 0.001).

Since BMVs are able to induce drastic effects on host cell metabolism, we assumed that other general cellular processes would also be impaired. To gain an overview of altered cellular functions, we performed RNA-sequencing analysis followed by pathway enrichment analysis (Fig. S5). We identified the suppression of cholesterol biosynthesis as an important target pathway in A549 cells treated with PA14 BMVs, as all participating genes were downregulated (Fig. 3A). Suppression of cholesterol biosynthesis is well documented in viral infections (36), but less explored in bacterial infections. For further validation, we analyzed the expression of selected downregulated genes by using quantitative PCR (Figs. 3B and S6). We observed a decreased expression of 3-hydroxy-3-methylglutaryl-coenzyme A reductase (HMGCR) and sterol regulatory element binding transcription factor 2 (SREBF2) after 24 h of BMV exposure. HMGCR is the rate-limiting enzyme of the cholesterol synthesis pathway (37), and its expression can be activated by SREBF2 which regulates various key enzymes associated with cholesterol and fatty acid synthesis (38, 39).

We also observed an upregulation of markers associated with inflammation (TNF signaling) and disease (lung fibrosis) after BMV treatment (Fig. 3A). For example, we determined a higher expression of interleukin-8 (IL-8, *CXCL8*), which plays a key role in acute inflammation (40). Bauman and Kuehn have previously shown that vesicles isolated from the *P. aeruginosa* PAO1 strain induce IL-8 activation in lung epithelial cells (7). To confirm this result using PA14 BMVs, we exposed lung cancer cells (A549) and primary lung cells (HBEpC) to BMVs for 24 h and measured their IL-8 secretion (Fig. 3C). We observed an increased IL-8 secretion for both cell lines, with a more pronounced effect in A549 cells, which could be explained by the general importance of IL-8 in cancer progression (41). Next, we analyzed whether vesicles affect not only transcription, but also translation in the host cell, since there are known bacterial regulators of protein translation, such as the exotoxin A of *P. aeruginosa* (42). Moreover, inhibition of protein synthesis by BMVs has already been described in macrophages due to mitochondrial stress (13). To confirm similar changes in human lung cells, we labeled newly translated proteins of A549 cells with a fluorescent dye and analyzed their signal intensities after treatment with the translation inhibitor cycloheximide (CHX) and PA14 BMVs. We observed a decreased fluorescence signal for both treatments already after 30 min, indicating the inhibition of global protein synthesis by PA14 BMVs in A549 cells (Fig. 3D).

### *P. aeruginosa* BMVs activate AMPK signaling through mitochondrial dysfunction, leading to global protein synthesis inhibition

We showed that PA14 BMVs are able to affect general cellular functions of the host cell, suggesting the activation of a global signaling pathway inside the cell. The AMPK is known to function as a metabolic sensor that can be activated e.g. by mitochondrial dysfunction, sensing decreased ATP levels caused by ETC inhibition (43). Moreover, mitochondria-localized AMPK is known to enable mitochondrial function (44). Losier et al. (10) demonstrated that AMPK is stimulated by the detection of BMVs during infection with the pathogen *Salmonella enterica* serovar Typhimurium, resulting in autophagy induction. Interestingly, AMPK activation is known to downregulate HMGCR, which in turn suppresses cholesterol synthesis (45), aligning with our result that HMGCR gene expression is decreased after BMV treatment (Fig. 3A and B).

**Fig. 4.**
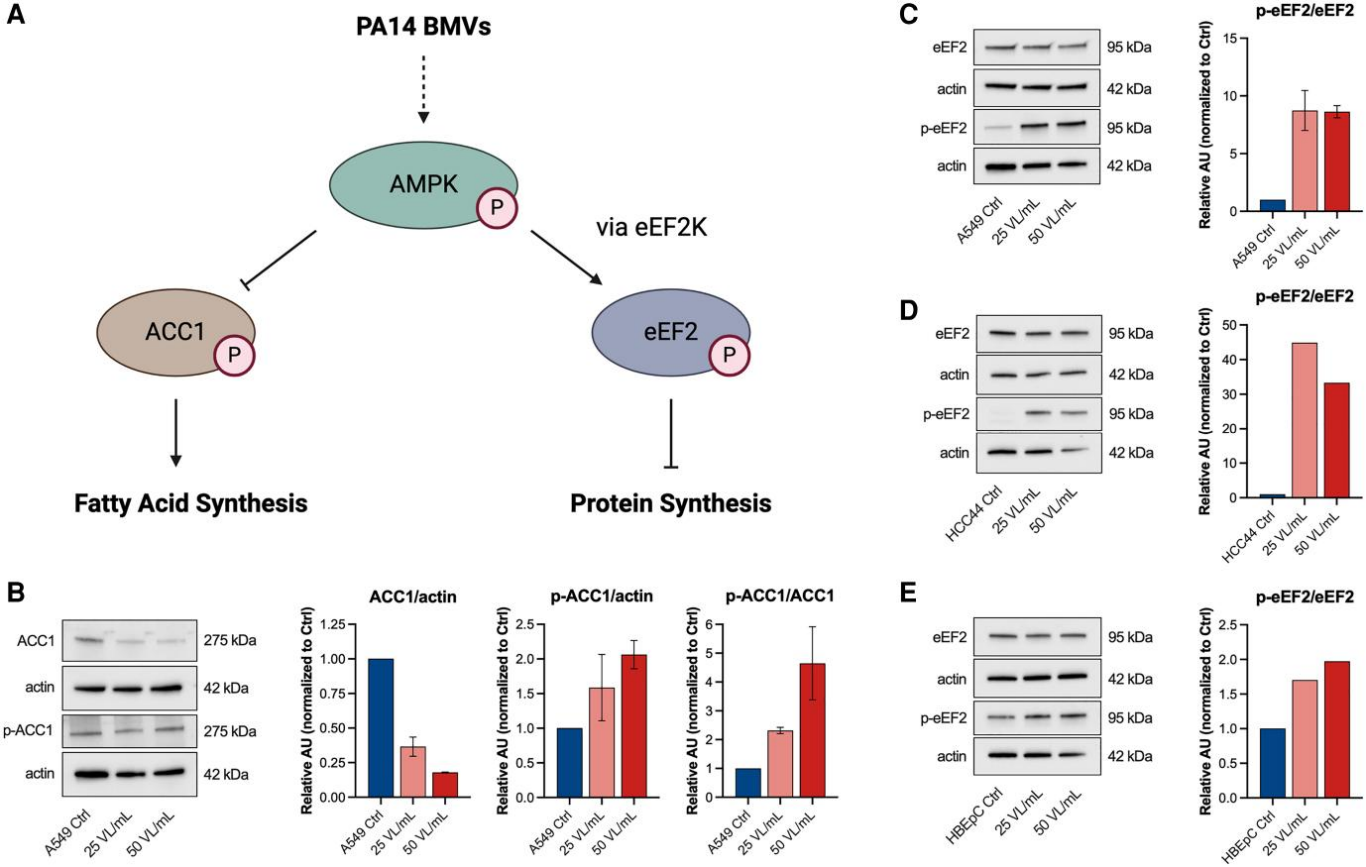
Activation of AMPK signaling in host cells by PA14 BMVs. A) Overview of the AMPK signaling targets ACC1 and eEF2 and their activation. B) Western blot (WB) analysis of p-ACC1 (Ser79) in A549 cells after vesicle treatment (25 and 50 VL/mL) for 24 h. Data were obtained from two independent experiments. C) WB analysis of p-eEF2 (Thr56) in A549 cells after vesicle treatment (25 and 50 VL/mL) for 24 h. Data were obtained from two independent experiments. D) WB analysis of p-eEF2 (Thr56) in HCC44 cells after vesicle treatment (25 and 50 VL/mL) for 24 h. E) WB analysis of p-eEF2 (Thr56) in HBEpC cells after vesicle treatment (25 and 50 VL/mL) for 24 h. All bar plots in this figure are depicted as mean (±SEM).

To confirm whether AMPK signaling is activated by PA14 BMVs, we treated A549 cells with *P. aeruginosa* BMVs and analyzed the activation of the AMPK target enzyme acetyl-CoA carboxylase 1 (ACC1, Fig. 4A) by determining its phosphorylation via western blot (WB) analysis. When phosphorylated, ACC1 inhibits the conversion of acetyl-CoA to malonyl-CoA, which is needed for fatty acid synthesis (46). We observed a general decrease in ACC1 protein levels after vesicle treatment, while the p-ACC1/ACC1 ratio increased in a concentration-dependent manner, indicating the activation of AMPK by PA14 BMVs (Fig. 4B). This result also suggests a regulation of ACC1 protein translation by BMVs, aligning with our observation that PA14 BMVs inhibit global protein synthesis in the host cell (Fig. 3D). Since AMPK signaling can regulate protein translation via the eukaryotic elongation factor 2 (eEF2), we analyzed its activation by WB analysis and observed a highly increased p-eEF2/eEF2 ratio after BMV treatment (Fig. 4C). As eEF2 acts as a negative regulator of protein synthesis (47), its activation indicates an inhibition of global protein synthesis by PA14 BMVs. We observed the activation of eEF2 in lung cancer cells (Fig. 4C and D) as well as in primary lung cells (Fig. 4E), with a higher phosphorylation increase in cancer cells.

Taken together, our results suggest that PA14 BMVs inhibit global protein synthesis in both lung cancer and primary lung cells, with a more pronounced effect in cancer cells.

**Fig. 5.**
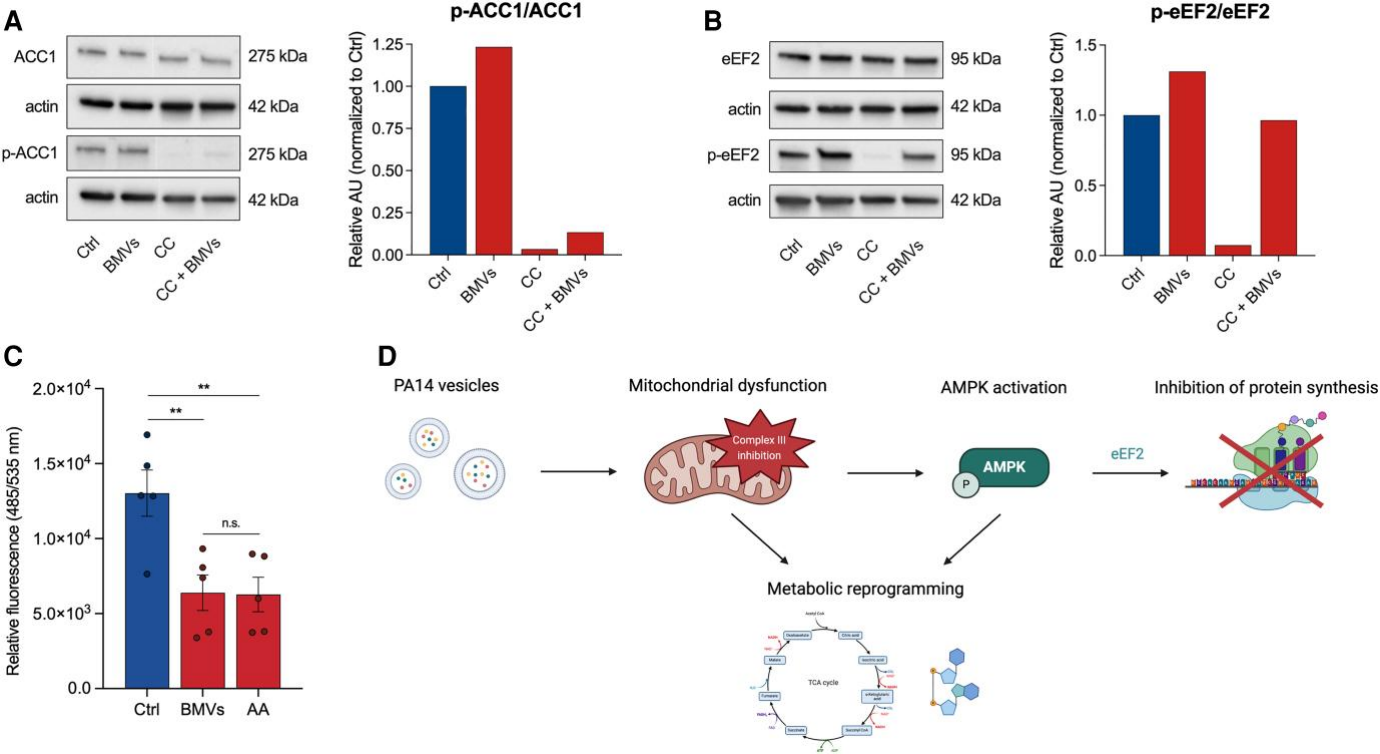
AMPK-mediated inhibition of global protein synthesis through ETC inhibition after PA14 BMV treatment. A) WB analysis of p-ACC1 (Ser79) in A549 cells after treatment with 25 VL/mL BMVs and 10 µM CC for 24 h. B) WB analysis of p-eEF2 (Thr56) in A549 cells after treatment with 25 VL/mL BMVs and 10 µM CC for 24 h. C) Global protein synthesis of A549 cells after treatment with 25 VL/mL BMVs or 1 µM AA for 30 min (+30 min pretreatment). Data were obtained from five replicates. D) Overview of the effects of PA14 BMVs on the host cell and their underlying signaling pathway. Vesicles specifically inhibit complex III of the ETC resulting in mitochondrial dysfunction which activates the AMPK signaling pathway leading to an AMPK-dependent inhibition of protein synthesis via the translation regulator eEF2. Mitochondrial dysfunction and AMPK activation both induce a metabolic reprogramming of the host cell. Created with BioRender.com. All bar plots in this figure are depicted as mean (±SEM). Statistical significance was analyzed using unpaired one-way ANOVA followed by Tukey's multiple comparison test (C) (n.s. = not significant, **P < 0.01).

Since it has also been shown that BMVs of *Salmonella enterica* serovar Typhimurium inhibit mTOR signaling (10), and given that eEF2 can also be regulated by mTOR, we analyzed the activity of the upstream kinase eEF2K, using an antibody for the mTOR-specific phosphorylation site Ser366 (47, 48). However, we did not observe any change in phosphorylation at this site (Fig. S7), suggesting that the vesicle-driven effects on protein synthesis are regulated independently of mTOR. To confirm that translation is inhibited via AMPK signaling, we treated BMV-infected A549 cells with the AMPK inhibitor compound C (CC) and analyzed the activity of ACC1 and eEF2. We discovered that AMPK inhibition suppressed the vesicle-mediated activation of both targets (Fig. 5A and B), connecting the inhibition of protein synthesis by BMVs to the activation of AMPK signaling.

As mentioned, AMPK is known to sense mitochondrial dysfunction caused by ETC inhibition. Toyama et al. (43) have already shown that AA, a commonly used inhibitor of complex III of the ETC, can rapidly activate AMPK, leading to mitochondrial fragmentation. A similar connection between mitochondrial dysfunction and AMPK activation is observed with the common type 2 diabetes drug metformin. Metformin inhibits complex I of the ETC and activates AMPK signaling, resulting in decreased cell proliferation and protein synthesis (49).

Since BMVs and AA both target complex III of the ETC, we wanted to test whether this impairment results in an inhibition of host cell protein synthesis. To that end, we treated A549 cells with PA14 BMVs and AA and analyzed global protein synthesis. We observed a similar inhibitory effect on global protein synthesis for both treatments (Fig. 5C).

Taken together, our results suggest that PA14 BMVs mediate mitochondrial dysfunction in the host cell due to the specific inhibition of complex III of the ETC, which then activates AMPK signaling, leading to AMPK-dependent inhibition of protein synthesis (Fig. 5D).

## Conclusion

In this study, we investigated the effects of *P. aeruginosa* PA14 BMVs on cell metabolism and mitochondrial respiration in human lung cells. In summary, we observed cellular metabolic reprogramming following vesicle treatment, particularly affecting TCA cycle-associated metabolites. In agreement with recent investigations (13), we identified vesicle-driven mitochondrial dysfunction, but more specifically, inhibition of complex III of the mitochondrial ETC by PA14 BMVs. We further demonstrated that BMV-mediated activation of the metabolic sensor AMPK (10) is presumably a consequence of impaired complex III function, and identified AMPK-dependent inhibition of global protein synthesis in the host cell. However, the findings presented in this study are limited by the reliance on in vitro experiments and the use of a single *P. aeruginosa* PA14 laboratory strain, which does not reflect the genetic diversity and virulence profiles of clinical isolates. To enhance the biological relevance and translational potential of the results, the use of primary cells from multiple donors and in vivo models should be considered in further studies. Despite these limitations, our study builds upon previously reported effects of BMVs on host cells, advancing this knowledge by uncovering a link between mitochondrial dysfunction and AMPK activation mediated by BMVs (10, 13).

## Supplementary Material

pgaf248_Supplementary_Data

## Data Availability

All data are included in the manuscript and Supplementary material. The RNA-Seq dataset (RNA-Seq PA14 A549) is available at https://doi.org/10.15490/FAIRDOMHUB.1.STUDY.1393.2.

## Preprints

A preprint of this article is published at https://doi.org/10.1101/2024.06.17.599317.

## References

[1] Bodey GP, Bolivar R, Fainstein V, Jadeja L. 1983. Infections caused by *Pseudomonas aeruginosa*. Rev Infect Dis. 5(2):279–313. 6405475 10.1093/clinids/5.2.279

[2] Stryjewski ME, Sexton DJ. *Pseudomonas aeruginosa* infections in specific types of patients and clinical settings. In: Hauser AR, Rello J, editors. Severe infections caused by Pseudomonas aeruginosa. Perspectives on critical care infectious diseases. Springer, Boston, MA, 2003. p. 1–15.

[3] Kadurugamuwa JL, Beveridge TJ. 1995. Virulence factors are released from *Pseudomonas aeruginosa* in association with membrane vesicles during normal growth and exposure to gentamicin: a novel mechanism of enzyme secretion. J Bacteriol. 177(14):3998–4008. 7608073 10.1128/jb.177.14.3998-4008.1995 PMC177130

[4] Beveridge TJ . 1999. Structures of gram-negative cell walls and their derived membrane vesicles. J Bacteriol. 181(16):4725–4733. 10438737 10.1128/jb.181.16.4725-4733.1999 PMC93954

[5] Sjöström AE, Sandblad L, Uhlin BE, Wai SN. 2015. Membrane vesicle-mediated release of bacterial RNA. Sci Rep. 5:15329. 26483327 10.1038/srep15329 PMC4612299

[6] Xie Z, et al 2024. *Pseudomonas aeruginosa* outer membrane vesicle-packed sRNAs can enter host cells and regulate innate immune responses. Microb Pathog. 188:106562. 38307370 10.1016/j.micpath.2024.106562

[7] Bauman SJ, Kuehn MJ. 2006. Purification of outer membrane vesicles from *Pseudomonas aeruginosa* and their activation of an IL-8 response. Microbes Infect. 8(9–10):2400–2408. 16807039 10.1016/j.micinf.2006.05.001 PMC3525494

[8] Ellis TN, Leiman SA, Kuehn MJ. 2010. Naturally produced outer membrane vesicles from *Pseudomonas aeruginosa* elicit a potent innate immune response via combined sensing of both lipopolysaccharide and protein components. Infect Immun. 78(9):3822–3831. 20605984 10.1128/IAI.00433-10 PMC2937433

[9] Takahara M, Hirayama S, Futamata H, Nakao R, Tashiro Y. 2024. Biofilm-derived membrane vesicles exhibit potent immunomodulatory activity in *Pseudomonas aeruginosa* PAO1. Microbiol Immunol. 68(7):224–236. 38797913 10.1111/1348-0421.13156

[10] Losier TT, et al 2019. AMPK promotes xenophagy through priming of autophagic kinases upon detection of bacterial outer membrane vesicles. Cell Rep. 26(8):2150–2165.e5. 30784596 10.1016/j.celrep.2019.01.062

[11] Zhao B, et al 2016. Mitochondrial dysfunction activates the AMPK signaling and autophagy to promote cell survival. Genes Dis. 3(1):82–87. 28066797 10.1016/j.gendis.2015.12.002 PMC5215801

[12] Han SY, et al 2018. Mitochondrial dysfunction induces the invasive phenotype, and cell migration and invasion, through the induction of AKT and AMPK pathways in lung cancer cells. Int J Mol Med. 42(3):1644–1652. 29916527 10.3892/ijmm.2018.3733

[13] Deo P, et al 2020. Mitochondrial dysfunction caused by outer membrane vesicles from gram-negative bacteria activates intrinsic apoptosis and inflammation. Nat Microbiol. 5(11):1418–1427. 32807891 10.1038/s41564-020-0773-2

[14] Escoll P, Platon L, Buchrieser C. 2019. Roles of mitochondrial respiratory complexes during infection. Immunometabolism. 1(2):e190011.

[15] Armstrong AV, Stewart-Tull DES. 1971. The site of the activity of extracellular products of *Pseudomonas aeruginosa* in the electron-transport chain in mammalian cell respiration. J Med Microbiol. 4(2):263–270. 4328182 10.1099/00222615-4-2-263

[16] Armstrong AV, Stewart-Tull DES, Roberts JS. 1971. Characterisation of the *Pseudomonas aeruginosa* factor that inhibits mouse–liver mitochondrial respiration. J Med Microbiol. 4(2):249–262. 4998856 10.1099/00222615-4-2-249

[17] Stewart-Tull DE, Armstrong AV. 1972. The effect of 1-hydroxyphenazine and pyocyanin from *Pseudomonas aeruginosa* on mammalian cell respiration. J Med Microbiol. 5(1):67–73. 4623349 10.1099/00222615-5-1-67

[18] Pavlovskis OR . 1972. *Pseudomonas aeruginosa* exotoxin: effect on cellular and mitochondrial respiration. J Infect Dis. 126(1):48–53. 4624886 10.1093/infdis/126.1.48

[19] Rahme LG, et al 1995. Common virulence factors for bacterial pathogenicity in plants and animals. Science. 268(5219):1899–1902. 7604262 10.1126/science.7604262

[20] Bagdasarian M, et al 1981. Specific-purpose plasmid cloning vectors. II. Broad host range, high copy number, RSF1010-derived vectors, and a host–vector system for gene cloning in *Pseudomonas*. Gene. 16(1–3):237–247. 6282695 10.1016/0378-1119(81)90080-9

[21] Kretschmer M, et al 2023. Isolation and quantification of bacterial membrane vesicles for quantitative metabolic studies using mammalian cell cultures. Cells. 12(23):2674. 38067103 10.3390/cells12232674 PMC10705164

[22] Sapcariu SC, et al 2014. Simultaneous extraction of proteins and metabolites from cells in culture. MethodsX. 18(1):74–80. 10.1016/j.mex.2014.07.002 PMC4472845 26150938

[23] Hiller K, et al 2009. MetaboliteDetector: comprehensive analysis tool for targeted and nontargeted GC/MS based metabolome analysis. Anal Chem. 81(9):3429–3439. 19358599 10.1021/ac802689c

[24] Jaber SM, Yadava N, Polster BM. 2020. Mapping mitochondrial respiratory chain deficiencies by respirometry: beyond the Mito Stress Test. Exp Neurol. 328:113282. 32165258 10.1016/j.expneurol.2020.113282 PMC7202675

[25] Ismail S, Hampton MB, Keenan JI. 2003. *Helicobacter pylori* outer membrane vesicles modulate proliferation and interleukin-8 production by gastric epithelial cells. Infect Immun. 71(10):5670–5675. 14500487 10.1128/IAI.71.10.5670-5675.2003 PMC201067

[26] Bartruff JB, Yukna RA, Layman DL. 2005. Outer membrane vesicles from *Porphyromonas gingivalis* affect the growth and function of cultured human gingival fibroblasts and umbilical vein endothelial cells. J Periodontol. 76(6):972–979. 15948693 10.1902/jop.2005.76.6.972

[27] Chen X, Zhang J, Yang M, Du G, Chen F. 2022. Methicillin-resistant *Staphylococcus aureus* membrane vesicles inhibit the proliferation and induce the apoptosis of epithelial cells. Pathogens. 11(12):1429. 36558763 10.3390/pathogens11121429 PMC9781941

[28] O’Neill LAJ, Kishton RJ, Rathmell J. 2016. A guide to immunometabolism for immunologists. Nat Rev Immunol. 16(9):553–565. 27396447 10.1038/nri.2016.70 PMC5001910

[29] Escoll P, Buchrieser C. 2018. Metabolic reprogramming of host cells upon bacterial infection: why shift to a Warburg-like metabolism? FEBS J. 285(12):2146–2160. 29603622 10.1111/febs.14446

[30] Goyal P, Rajala MS. 2023. Reprogramming of glucose metabolism in virus infected cells. Mol Cell Biochem. 478(11):2409–2418. 36709223 10.1007/s11010-023-04669-4 PMC9884135

[31] Filipp FV, Scott DA, Ronai ZA, Osterman AL, Smith JW. 2012. Reverse TCA cycle flux through isocitrate dehydrogenases 1 and 2 is required for lipogenesis in hypoxic melanoma cells. Pigment Cell Melanoma Res. 25(3):375–383. 22360810 10.1111/j.1755-148X.2012.00989.x PMC3329592

[32] Zaidi N, Swinnen JV, Smans K. 2012. ATP-citrate lyase: a key player in cancer metabolism. Cancer Res. 72(15):3709–3714. 22787121 10.1158/0008-5472.CAN-11-4112

[33] Lao-On U, Attwood PV, Jitrapakdee S. 2018. Roles of pyruvate carboxylase in human diseases: from diabetes to cancers and infection. J Mol Med. 96(3–4):237–247. 29362846 10.1007/s00109-018-1622-0

[34] Lampropoulou V, et al 2016. Itaconate links inhibition of succinate dehydrogenase with macrophage metabolic remodeling and regulation of inflammation. Cell Metab. 24(1):158–166. 27374498 10.1016/j.cmet.2016.06.004 PMC5108454

[35] Cordes T, Metallo CM. 2021. Itaconate alters succinate and coenzyme A metabolism via inhibition of mitochondrial complex II and methylmalonyl-CoA mutase. Metabolites. 11(2):117. 33670656 10.3390/metabo11020117 PMC7922098

[36] Blanc M, et al 2011. Host defense against viral infection involves interferon mediated down-regulation of sterol biosynthesis. PLoS Biol. 9(3):e1000598. 21408089 10.1371/journal.pbio.1000598 PMC3050939

[37] Lindgren V, Luskey KL, Russell DW, Francke U. 1985. Human genes involved in cholesterol metabolism: chromosomal mapping of the loci for the low density lipoprotein receptor and 3-hydroxy-3-methylglutaryl-coenzyme A reductase with cDNA probes. Proc Natl Acad Sci U S A. 82(24):8567–8571. 3866240 10.1073/pnas.82.24.8567 PMC390958

[38] Hua X, et al 1993. SREBP-2, a second basic-helix-loop-helix-leucine zipper protein that stimulates transcription by binding to a sterol regulatory element. Proc Natl Acad Sci U S A. 90(24):11603–11607. 7903453 10.1073/pnas.90.24.11603 PMC48032

[39] Madison BB . 2016. Srebp2: a master regulator of sterol and fatty acid synthesis. J Lipid Res. 57(3):333–335. 26798145 10.1194/jlr.C066712 PMC4766982

[40] Harada A, et al 1994. Essential involvement of interleukin-8 (IL-8) in acute inflammation. J Leukoc Biol. 56:559–564. 7964163

[41] Liu Q, et al 2016. The CXCL8-CXCR1/2 pathways in cancer. Cytokine Growth Factor Rev. 31:61–71. 27578214 10.1016/j.cytogfr.2016.08.002 PMC6142815

[42] Saelinger CB . 1988. Use of exotoxin A to inhibit protein synthesis. Methods Enzymol. 165:226–231. 3148096 10.1016/s0076-6879(88)65035-x

[43] Toyama EQ, et al 2016. Metabolism. AMP-activated protein kinase mediates mitochondrial fission in response to energy stress. Science. 351(6270):275–281. 26816379 10.1126/science.aab4138 PMC4852862

[44] Drake JC, et al 2021. Mitochondria-localized AMPK responds to local energetics and contributes to exercise and energetic stress-induced mitophagy. Proc Natl Acad Sci U S A. 118(37):e2025932118. 34493662 10.1073/pnas.2025932118 PMC8449344

[45] Loh K, et al 2018. Inhibition of adenosine monophosphate-activated protein kinase-3-hydroxy-3-methylglutaryl coenzyme A reductase signaling leads to hypercholesterolemia and promotes hepatic steatosis and insulin resistance. Hepatol Commun. 3(1):84–98. 30619997 10.1002/hep4.1279 PMC6312662

[46] Fullerton MD, et al 2013. Single phosphorylation sites in acc1 and acc2 regulate lipid homeostasis and the insulin-sensitizing effects of metformin. Nat Med. 19(12):1649–1654. 24185692 10.1038/nm.3372 PMC4965268

[47] Johanns M, et al 2017. Direct and indirect activation of eukaryotic elongation factor 2 kinase by AMP-activated protein kinase. Cell Signal. 36:212–221. 28502587 10.1016/j.cellsig.2017.05.010

[48] Wang X, et al 2001. Regulation of elongation factor 2 kinase by p90*^RSK^*^1^ and p70 S6 kinase. EMBO J. 20(16):4370–4379. 11500364 10.1093/emboj/20.16.4370 PMC125559

[49] Tosca L, Ramé C, Chabrolle C, Tesseraud S, Dupont J. 2010. Metformin decreases IGF1-induced cell proliferation and protein synthesis through AMP-activated protein kinase in cultured bovine granulosa cells. Reproduction. 139(2):409–418. 19906888 10.1530/REP-09-0351

