# Supplementary material for: Bacterial membrane vesicles of Pseudomonas aeruginosa activate adenosine monophosphate-activated protein kinase signaling through inhibition of mitochondrial complex III
Source: PNAS Nexus. 2025 Aug 19;4(8):pgaf248. doi: 10.1093/pnasnexus/pgaf248 (PMC12368959; doi:10.1093/pnasnexus/pgaf248)
Supplement: pgaf248_Supplementary_Data [file pgaf248_supplementary_data.docx]

BACTERIAL MEMBRANE VESICLES OF *PSEUDOMONAS AERUGINOSA* ACTIVATE AMPK SIGNALING THROUGH INHIBITION OF MITOCHONDRIAL COMPLEX III

Julia Müller^1,2^, Marcel Kretschmer^1,2^, Elise Opitsch^1,2^, Svea Holland^1,3^, José Manuel Borrero-de Acuña^4^, Dieter Jahn^1,5,*^, Meina Neumann-Schaal^1,3;*^, and Andre Wegner^1,2^

1Braunschweig Integrated Centre of Systems Biology (BRICS), Technische Universität Braunschweig, Rebenring 56, 38106 Braunschweig, Germany

2Department of Bioinformatics and Biochemistry, Technische Universität Braunschweig, Rebenring 56, 38106

Braunschweig, Germany

3Leibniz Institute DSMZ - German Collection of Microorganisms and Cell Cultures GmbH, Inhoffenstraße 7B, 38124 Braunschweig, Germany

4Departamento de Microbiología, Facultad de Biología, Universidad de Sevilla, Av. de la Reina Mercedes 6,

41012 Sevilla, Spain

5Institute for Microbiology, Technische Universität Braunschweig, 38106 Braunschweig, Germany

# *∗*Corresponding Authors

Dieter Jahn

E-Mail: [d.jahn@tu-braunschweig.de](mailto:d.jahn@tu-bs.de)

Meina Neumann-Schaal

E-Mail: [meina.neumann-schaal@dsmz.de](mailto:meina.neumann-schaal@dsmz.de)

**Keywords**: bacterial membrane vesicles (BMVs) | membrane vesicles (MVs) | outer membrane vesicles (OMVs) | *Pseudomonas aeruginosa* | pathogen | metabolism | cholesterol | mitochondria | respiration | electron transport chain | AMPK | protein synthesis

# SUPPLEMENTARY FIGURES


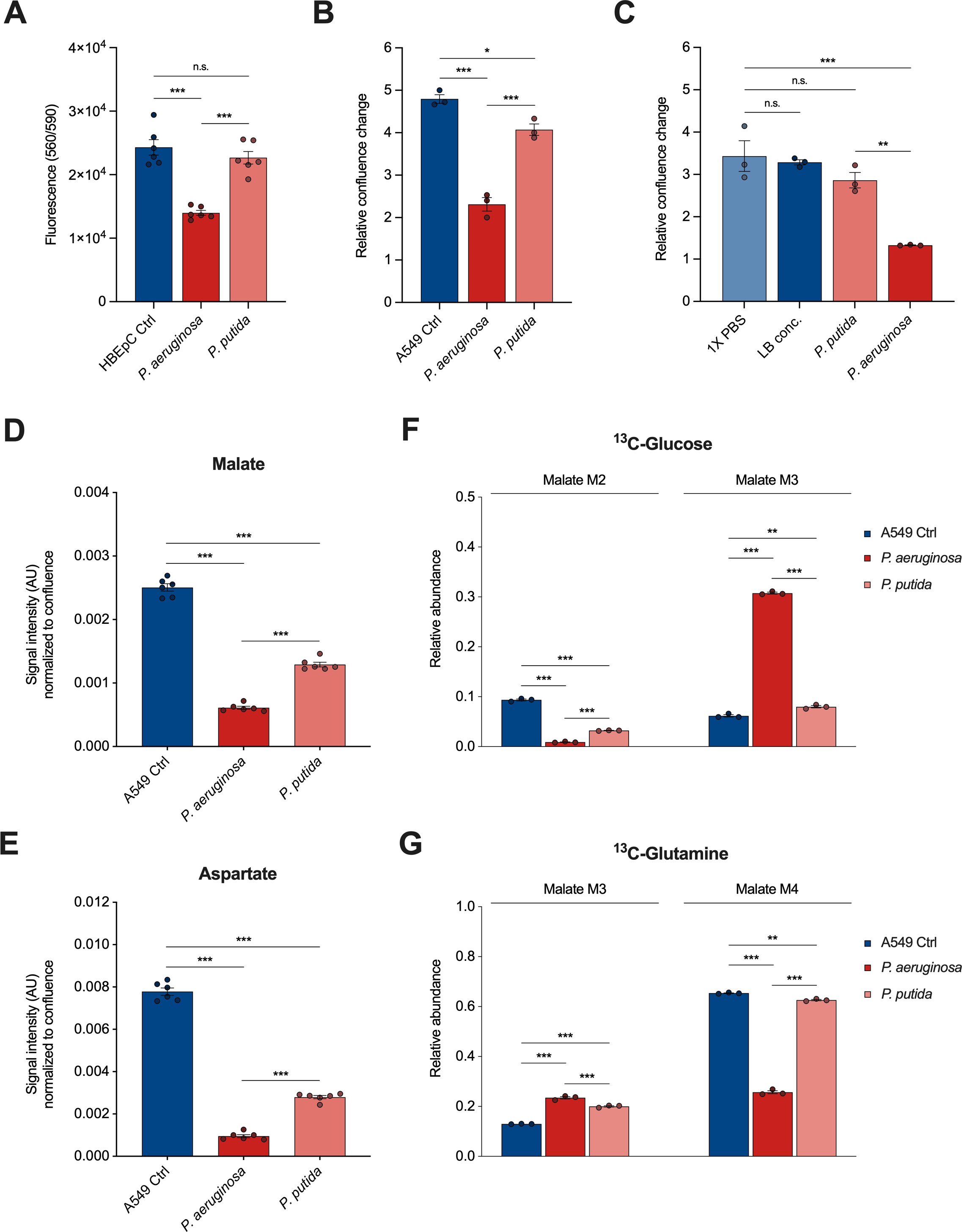


**Figure S1: Reduced effects of BMVs isolated from the non-pathogenic strain *P. putida* KT2440 compared to *P. aeruginosa* PA14 BMVs. (A)** Cell viability of HBEpC cells after treatment with 25 VL/mL BMVs for 24 h. Data were obtained from 6 replicates. **(B)** Confluence of A549 cells after treatment with 25 VL/mL BMVs for 72 h. Data were obtained from 3 replicates. **(C)** Confluence of A549 cells after treatment with 1X PBS, concentrated LB medium or 25 VL/mL BMVs isolated from *P. putida* KT2440, or *P. aeruginosa* PA14 for 72 h. Data were obtained from 3 replicates. **(D)** Signal intensity (AU) of malate in A549 cells after vesicle treatment (25 VL/mL for 24 h). Data were obtained from 6 replicates and normalized to cell confluence. **(E)** Signal intensity (AU) of aspartate in A549 cells after vesicle treatment (25 VL/mL for 24 h). Data were obtained from 6 replicates and normalized to cell confluence. **(F)** Malate MIDs after [U-^13^C_6_]-glucose labeling of BMV-treated A549 cells (25 VL/mL for 24 h). Data were obtained from 3 replicates. **(G)** Malate MIDs after [U-^13^C_5_]-glutamine labeling of BMV-treated A549 cells (25 VL/mL for 24 h). Data were obtained from 3 replicates. All bar plots in this Figure are depicted as mean *±* SEM. Statistical significance was analyzed using unpaired one-way ANOVA followed by Tukey’s multiple comparison test (n.s. = not significant, * = *p <* 0.05, ** = *p <* 0.01, *** = *p*

*<* 0.001).


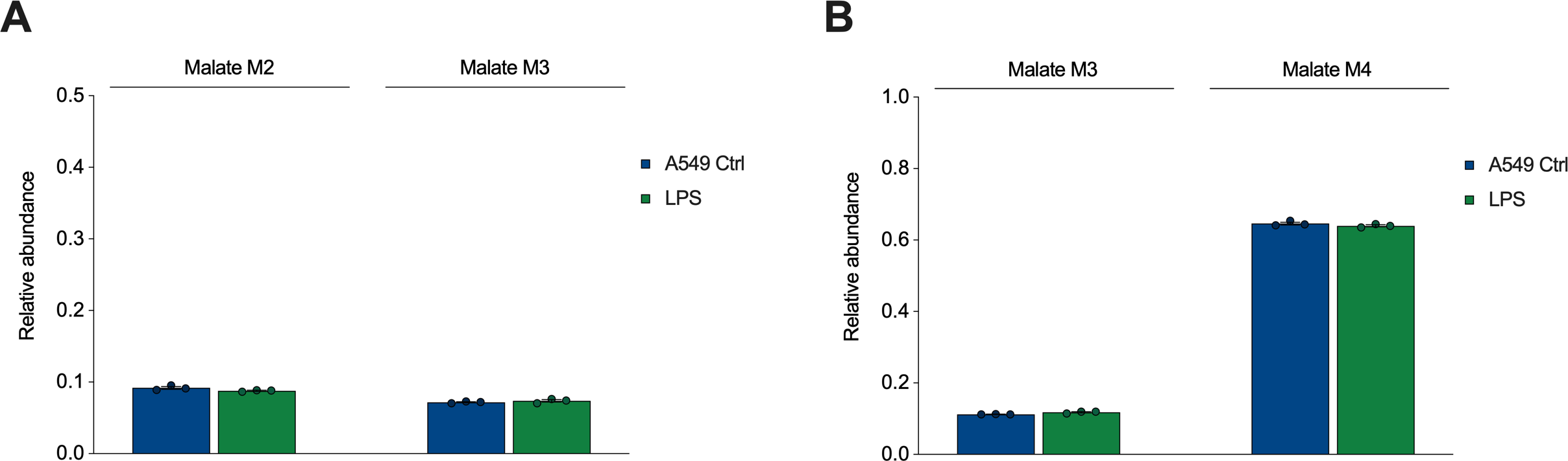


**Figure S2: Metabolic adaptations in A549 cells are not mediated by bacterial lipopolysaccharide (LPS).**

**(A)** Malate MIDs after [U-^13^C_6_]-glucose labeling of LPS-treated A549 cells (1 µg/mL for 24 h). Data were obtained from 3 replicates. **(B)** Malate MIDs after [U-^13^C_5_]-glutamine labeling of LPS-treated A549 cells (1 µg/mL for 24 h). Data were obtained from 3 replicates. Data were obtained from 3 replicates. All bar plots in this Figure are depicted as mean *±* SEM.


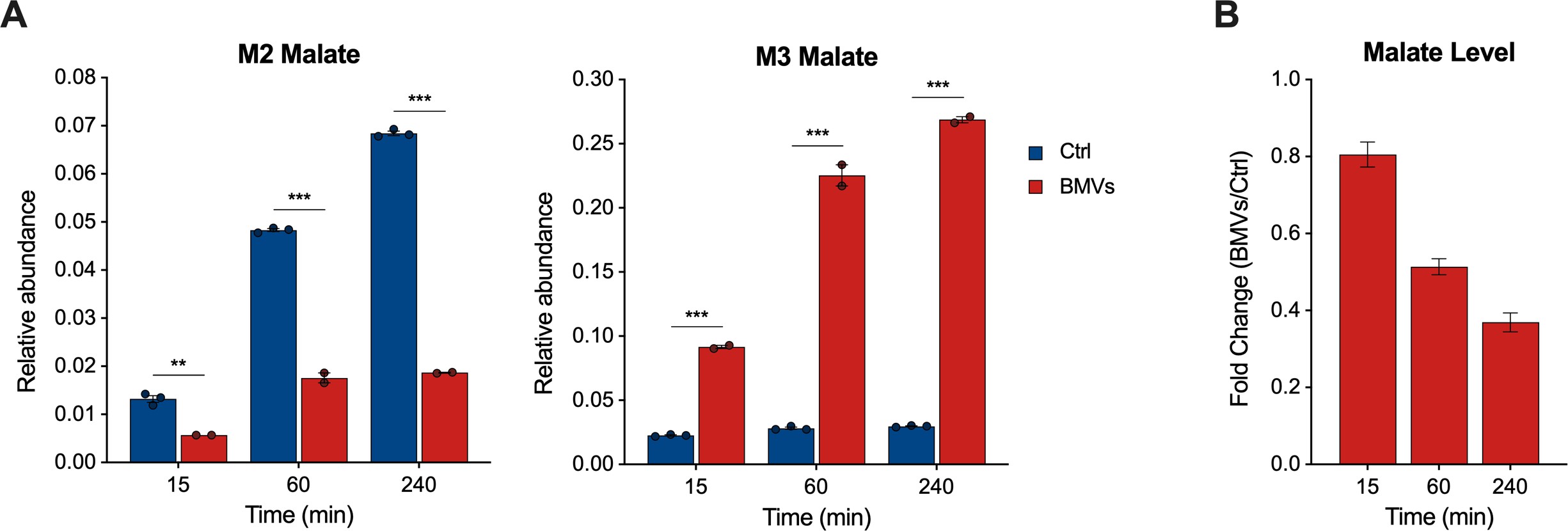


**Figure S3: Metabolic changes in A549 cells treated with *P. aeruginosa* PA14 BMVs after different timepoints. (A)** Malate MIDs of [U-^13^C_6_]-glucose labeled A549 cells after treatment with 25 VL/mL BMVs at three different timepoints (15, 60 and 240 min). Data were obtained from 2 or 3 replicates. **(B)** Relative signal intensities (fold change) of malate in BMV-treated A549 cells (25 VL/mL for 24 h) and cells without treatment (Ctrl). Data were obtained from 2 or 3 replicates. All bar plots in this Figure are depicted as mean *±* SEM. Statistical significance was analyzed using unpaired *t* -test (**A**) (** = *p <* 0.01, *** = *p <* 0.001).


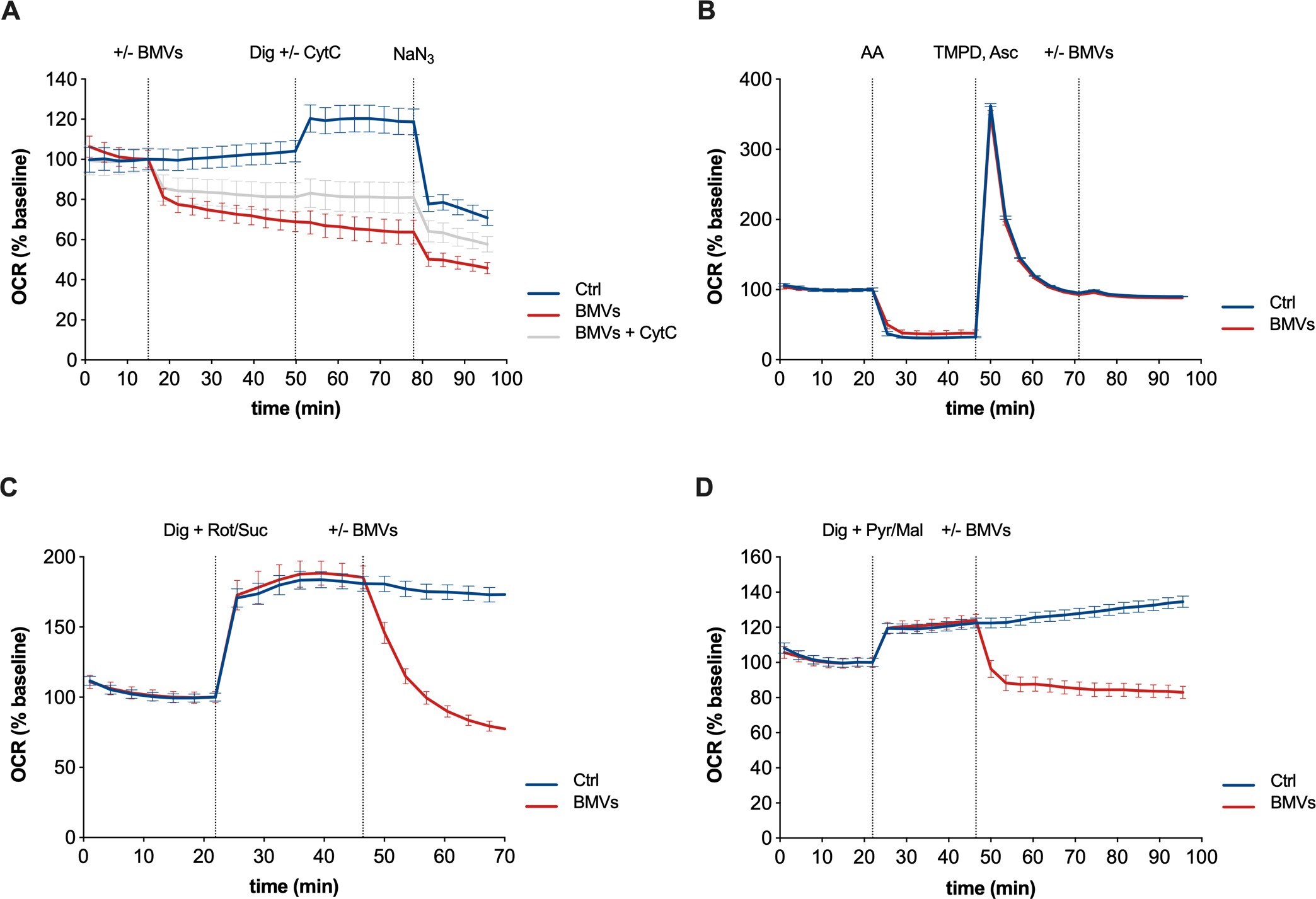


**Figure S4: Respirometry tests for the analysis of PA14 BMV-induced effects on the ETC of A549 cells.**

**(A)** Respirometry test for Cytochrome *c* (CytC) activity of A549 cells treated with 25 VL/mL BMVs. Cytochrome *c* delivery (100 µM, blue and gray line) to mitochondria was accomplished by addition of 25 µg/mL digitonin (Dig), together with 5 mM succinate, 1 mM ADP, 5 mM EDTA and 3.6 mM phosphate (K_2_HPO_4_). For complex IV inhibition, 5 mM sodium azide (NaN_3_) was added. Data were obtained from 20 replicates. **(B)** Respirometry test for complex IV activity of A549 cells treated with 25 VL/mL BMVs. Isolation of complex IV was performed by the addition of 0.5 µM Antimycin A (AA), 0.8 mM *N*,*N*,*N*’,*N*’-tetramethyl-*p*-phenylenediamine (TMPD) and 16 mM ascorbate (Asc). Data were obtained from 15 replicates. **(C)** Respirometry test for complex II activity of A549 cells treated with 25 VL/mL BMVs. Isolation of complex II was performed by the addition of 25 µg/mL digitonin (Dig), together with the substrates rotenone (0.5 µM) and succinate (5 mM), 0.3 mM ADP, 5 mM EDTA and 3.6 mM phosphate (K_2_HPO_4_). Data were obtained from 15 replicates. **(D)** Respirometry test for complex I activity of A549 cells treated with 25 VL/mL BMVs. Isolation of complex I was performed by the addition of 25 µg/mL digitonin (Dig), together with the substrates pyruvate (5 mM) and malate (5 mM), 0.3 mM ADP, 5 mM EDTA and 3.6 mM phosphate (K_2_HPO_4_). Data were obtained from 15 replicates. OCR values were normalized to the baseline and depicted as mean *±* SEM. Substances were added at the depicted time points.


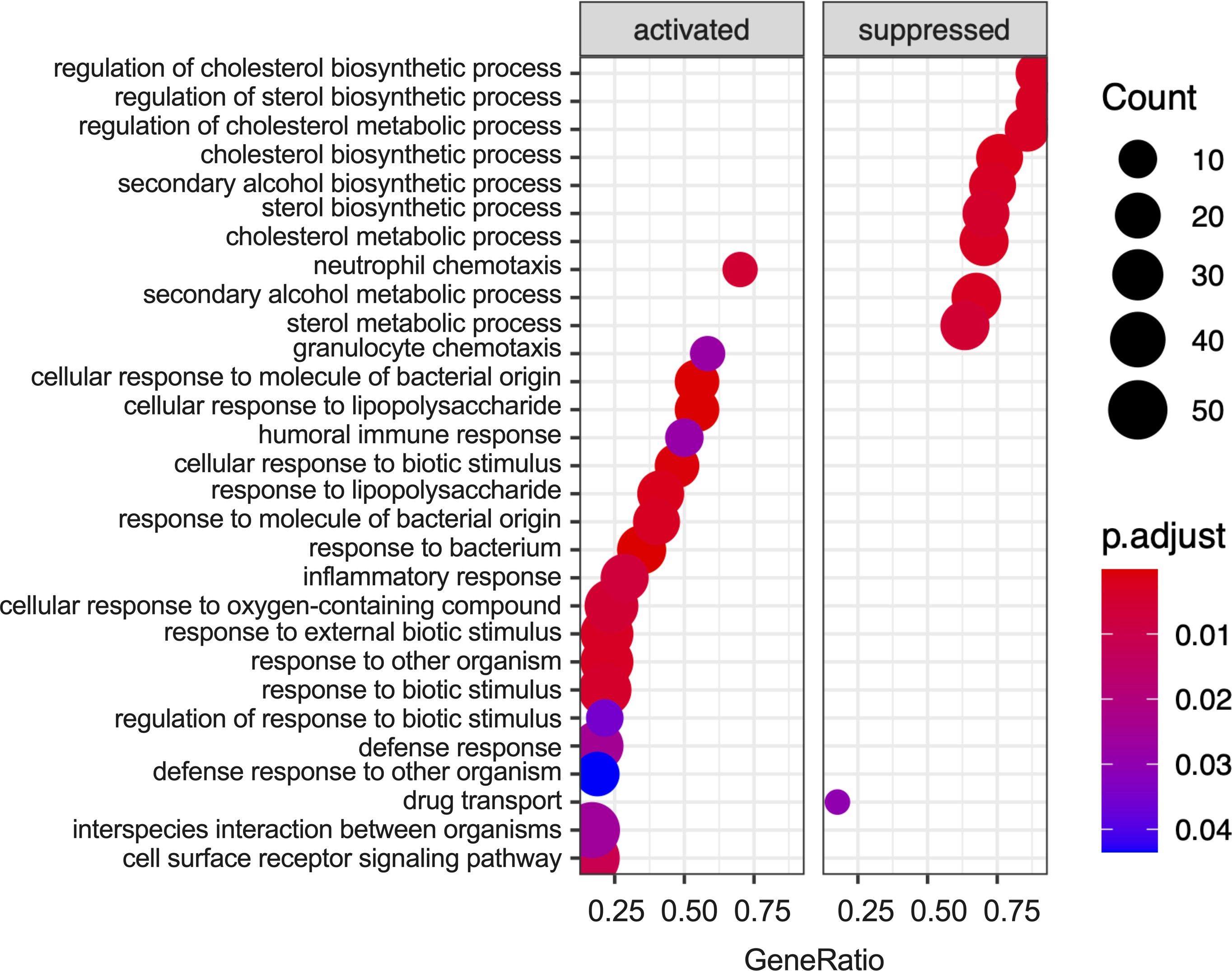


**Figure S5: Significantly enriched (activated and suppressed) GO-BP pathways in A549 cells following PA14 BMV treatment.** The vertical axis lists the names of GO-BP terms, while the length of the horizontal bars represents the gene ratio. The area of each circle indicates the gene counts, and the color intensity reflects the adjusted P-value. GO-BP: Gene Ontology biological process.


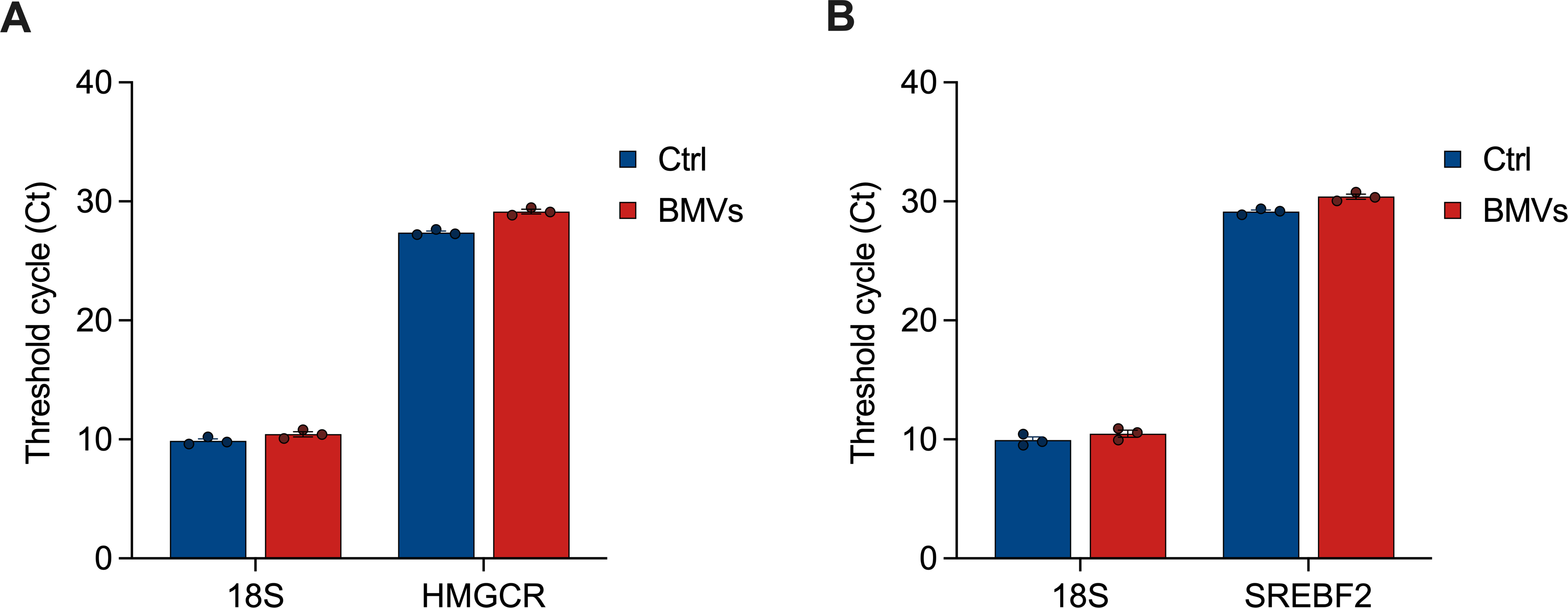


**Figure S6: Analysis of HMGCR and SREBF2 gene expression using qPCR.** Threshold cycle (Ct) values of HMGCR (**A**) and SREBF2 (**B**) in A549 cells after treatment with 25 VL/mL PA14 BMVs for 24 h. Data were obtained from 3 replicates.


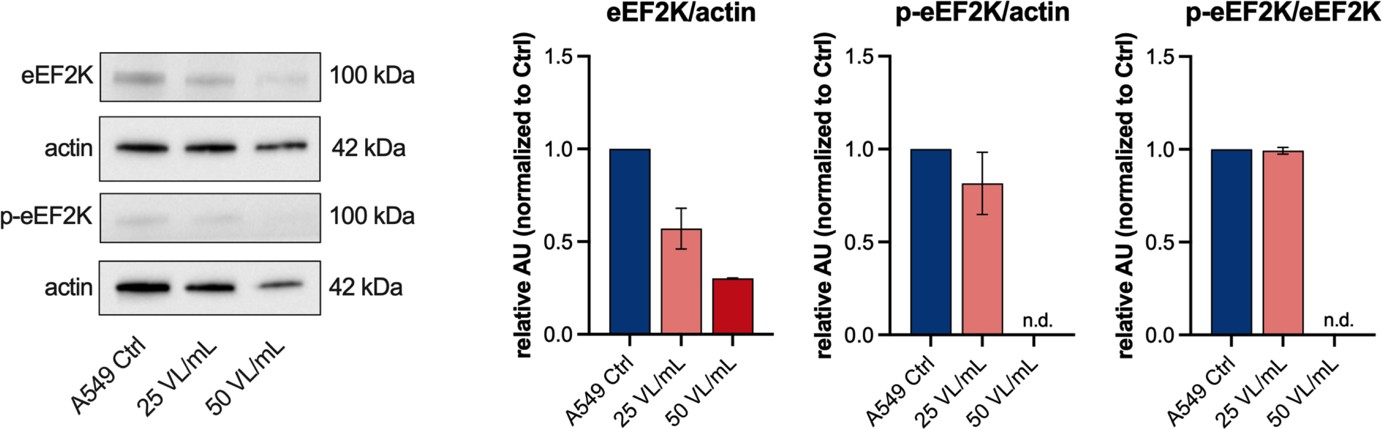


**Figure S7: Western blot analysis of the mTOR-specific phosphorylation site of eEF2K in A549 cells after PA14 BMV treatment.** WB analysis of p-eEF2K (Ser366) in A549 cells after vesicle treatment (25 VL/mL and 50 VL/mL) for 24 h. Data were obtained from 2 independent experiments. All bar plots in this Figure are depicted as mean (*±* SEM). n.d. = not detectable.

# SUPPLEMENTARY MATERIALS AND METHODS

1. **Analysis of uptake and secretion rates**
2. Glucose uptake and lactate secretion were measured by using the YSI 2950 biochemistry analyzer.
3. To that end, medium samples were collected before metabolite extraction, centrifuged at 4 °C and
4. 17,000 xg for 5 min and 200 µL of each sample was transferred in a 96-well microplate. For calibration,
5. a standard solution containing 28 mM glucose and 15 mM lactate was measured in a dilution series
6. of 5, 10, 20, 40, 60, 80 and 100 %.

# Analysis of cellular NADH, NAD+, and ATP levels

1. Cellular NADH and NAD+ levels were analyzed using the NAD/NADH-Glo™ Assay (Promega,
2. G9071) according to the user manual. For the analysis of cellular ATP levels, the CellTiter-Glo^®^
3. Luminescent Cell Viability Assay (Promega, G7570) was used. Briefly, cells were seeded into white
4. 96-well microplates (Greiner Bio-One, 655074) and incubated at 37 °C and 5 % CO2 for 24 h. The
5. next day, cells were washed with 1X PBS and treated as indicated in the figure legends. Following
6. incubation, ATP standard dilutions (0-1 µM) were added to the plate, which was then equilibrated at
7. room temperature for 30 min. For cell lysis, the prepared CellTiter-Glo^®^ reagent was added and the
8. plate mixed for 2 min using an orbital shaker. After an additional incubation at room temperature for
9. 10 min, luminscence intensities were measured using a microplate reader (Tecan Spark^®^).

# Measurement of interleukin-8 secretion

1. To analyze IL-8 secretion, cells were seeded in 6-well plates in growth medium and incubated at
2. 37 °C and 5 % CO2 overnight. The next day, cells were treated as indicated in the figure legends and
3. incubated at 37 °C and 5 % CO2 for 24 h. After treatment, medium was collected and centrifuged
4. at 4 °C and 17,000 x g for 5 min to remove cell debris. IL-8 concentration in medium samples was
5. measured by using the ELISA MAX Deluxe Set Human IL-8 (BioLegend, 431504) according to the
6. user manual.

**Table S1: Primary antibodies.**

| Primary antibody | Catalog no. | Company | Dilution |
| --- | --- | --- | --- |
| Mouse monoclonal anti-*β*-actin | A5441 | Merck | 1:5,000 |
| Rabbit polyclonal anti-ACC1 | 21923-1-AP | Proteintech | 1:4,000 |
| Rabbit polyclonal anti-phospho-ACC1 (Ser79) | 29119-1-AP | Proteintech | 1:1,000 |
| Rabbit polyclonal anti-eEF2K | 13510-1-AP | Proteintech | 1:1,000 |
| Rabbit polyclonal anti-phospho-eEF2K (Ser366) | 29032-1-AP | Proteintech | 1:2,000 |
| Rabbit polyclonal anti-eEF2 | 20107-1-AP | Proteintech | 1:10,000 |
| Rabbit polyclonal anti-phospho-eEF2 (Thr56) | E-AB-51049 | Elabscience | 1:1,000 |

# Analysis of global protein synthesis

1. For the analysis of global protein synthesis, cells were seeded in black 96-well microplates (Greiner
2. Bio-One, 655090) in growth medium and incubated at 37 °C and 5 % CO2 overnight. The next day,
3. treatments were performed as indicated in the figure legends. Protein synthesis of treated cells was
4. measured by using the Protein Synthesis Assay Kit (Cayman Chemical, 601100) according to the
5. user manual.

# RNA-Sequencing analysis

1. Cells were seeded in 6-well plates and incubated at 37 °C and 5 % CO2 overnight. The next day,
2. cells were washed with 1X PBS, treated with BMVs and incubated at 37 °C and 5 % CO2 for 24 h.
3. RNA isolation was performed by using the NucleoSpin^®^ RNA kit (MACHEREY-NAGEL, 740955.50)
4. as described above. cDNA synthesis was performed and libraries were constructed using NEBNext
5. Ultra II Directional RNA Library Prep Kit for Illumina at the Genome Analytics at Helmholtz Centre for
6. Infection Research. Libraries were sequenced using a NovaSeq 6000 instrument (Illumina) generating
7. 50-bp reads in paired-end mode. Reads mapping and differential expression analysis were performed
8. using the galaxy platform [(https://usegalaxy.org/)](https://usegalaxy.org/) [[1](#_bookmark0)]. Reads were mapped to the human genome
9. hg19 with Hisat2 [[2](#_bookmark1)]. EdgeR was used to identify differential expression and calculate the *P* values
10. with an exact test based on the dispersion generated by the quantile-adjusted conditional maximum
11. likelihood (qCML) method [[3].](#_bookmark2)

**Table S2: Secondary antibodies.**

| Secondary antibody | Catalog no. | Company | Dilution |
| --- | --- | --- | --- |
| Goat anti-mouse, HRP-conjugated | R-05071-500 | Advansta | 1:20,000 |
| Goat anti-rabbit, HRP-conjugated | R-05072-500 | Advansta | 1:20,000 |

# References

1. [1] Enis Afgan, Dannon Baker, Bérénice Batut, Marius Van Den Beek, Dave Bouvier, Martin Ech, John
2. Chilton, Dave Clements, Nate Coraor, Björn A. Grüning, Aysam Guerler, Jennifer Hillman-Jackson,
3. Saskia Hiltemann, Vahid Jalili, Helena Rasche, Nicola Soranzo, Jeremy Goecks, James Taylor,
4. Anton Nekrutenko, and Daniel Blankenberg. The Galaxy platform for accessible, reproducible and
5. collaborative biomedical analyses: 2018 update. *Nucleic Acids Research*, 46(W1):W537–W544,
6. 2018.
7. [2] Daehwan Kim, Ben Langmead, and Steven L. Salzberg. HISAT: a fast spliced aligner with low
8. memory requirements. *Nature Methods*, 12(4):357–360, 2015.
9. [3] Mark D. Robinson, Davis J. McCarthy, and Gordon K. Smyth. edgeR: a Bioconductor package for
10. differential expression analysis of digital gene expression data. *Bioinformatics*, 26(1):139–140,
11. 2010.
